# Supplementary material for: MetaQTL: a package of new computational methods for the meta-analysis of QTL mapping experiments
Source: BMC Bioinformatics. 2007 Feb 8;8:49. doi: 10.1186/1471-2105-8-49 (PMC1808479; doi:10.1186/1471-2105-8-49)
Supplement: Additional File 7 — MetaQTL Package : jar file and tutorial. This Zip archive contains both the MetaQTL JAR file and the files of the tutorial. [file 1471-2105-8-49-S7.zip › org.inra.metaqtl/doc/org/thalia/bio/entity/package-frame.html]

org.thalia.bio.entity


org.thalia.bio.entity

Interfaces 
  
*IBioConstants*
  
*IGeneticMapProperties*

Classes 
  
AlleleContainer
  
BioEntity
  
BioEntityCollection
  
BioEntityContainer
  
CrossPopulation
  
GeneticMap
  
GroupContainer
  
Individual
  
IndividualContainer
  
LGroup
  
Locus
  
LocusContainer
  
Marker
  
Ontology
  
OntologyTerm
  
Population
  
QTL
